# Publisher Correction: Vesicles driven by dynein and kinesin exhibit directional reversals without regulators

**DOI:** 10.1038/s41467-024-45256-5

**Published:** 2024-01-25

**Authors:** Ashwin I. D’Souza, Rahul Grover, Gina A. Monzon, Ludger Santen, Stefan Diez

**Affiliations:** 1 B CUBE - Center for Molecular Bioengineering, TU Dresden, Dresden Germany; 2 Center for Biophysics, Department of Physics, Saarland University, Saarbrücken, Germany; 3 Cluster of Excellence Physics of Life, TU Dresden, Dresden Germany; 4 Max Planck Institute of Molecular Cell Biology and Genetics, Dresden, Germany

**Keywords:** Dynein, Kinesin, Intracellular movement, Microtubules

Correction to: *Nature Communications* 10.1038/s41467-023-42605-8, published online 20 November 2023

The original version of this Article contained errors in Figures 2, 3, 5, and 6. In Fig. 2a, an extra line was inadvertently included. In Fig. 3a, some of the data lines were inadvertently shown as thicker than others. In Fig. 5a, arrows were missing the associated lines, and some arrowheads were pointing the wrong direction. In Fig. 6, arrows were missing the associated lines, and some arrowheads were pointing the wrong direction. This has been corrected in both the PDF and HTML versions of the Article.

