# Supplementary material for: Vesicles driven by dynein and kinesin exhibit directional reversals without regulators
Source: Nat Commun. 2023 Nov 20;14:7532. doi: 10.1038/s41467-023-42605-8 (PMC10662051; doi:10.1038/s41467-023-42605-8)
Supplement: Supplementary file 1 — Supplementary Information [file 41467_2023_42605_MOESM1_ESM.pdf]

Supplementary Information

Vesicles driven by dynein and kinesin exhibit directional reversals without regulators

Ashwin I. D'Souza, Rahul Grover, Gina A. Monzon, Ludger Santen and Stefan Diez

Supplementary Figures

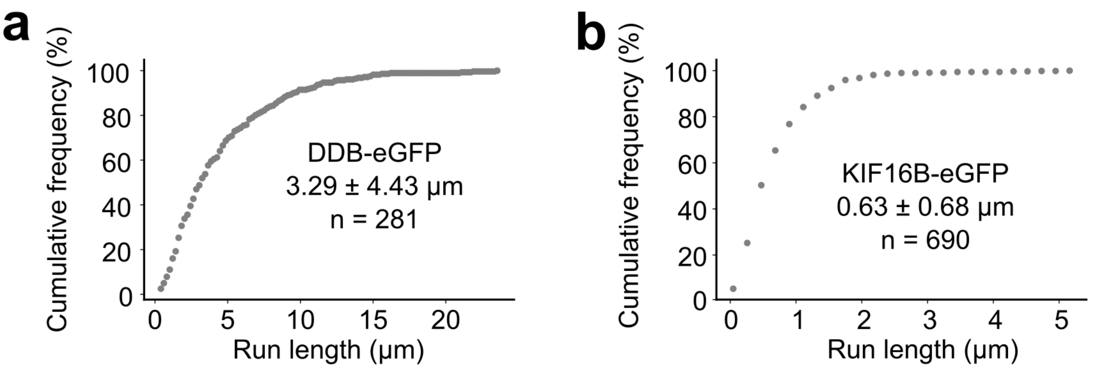

Supplementary Figure 1 | Purified DDB-eGFP complexes and KIF16B-eGFP are processive *in vitro*. Cumulative distribution frequency of run length of **a)** DDB-eGFP and **b)** KIF16B-eGFP. Numerical values are reported as median ± IQR. n represents the number of single-molecules/complexes.

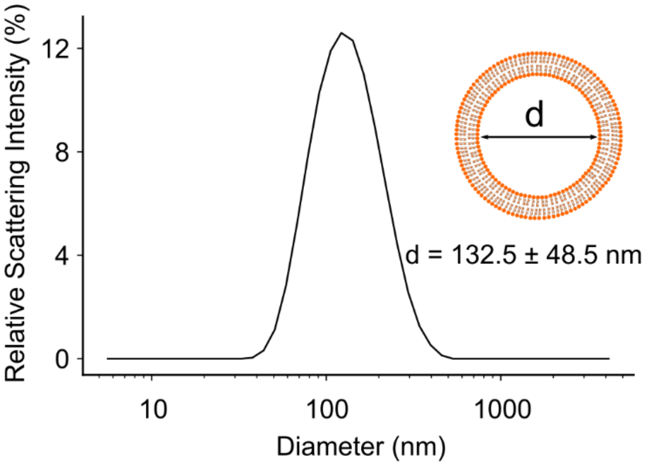

Supplementary Figure 2 | Uniformly sized vesicles are obtained by extrusion. Size distribution of large unilamellar vesicles obtained by dynamic light scattering (Zetasizer, Malvern). The size histogram was obtained from a single DLS run. d is reported as mean ± standard deviation

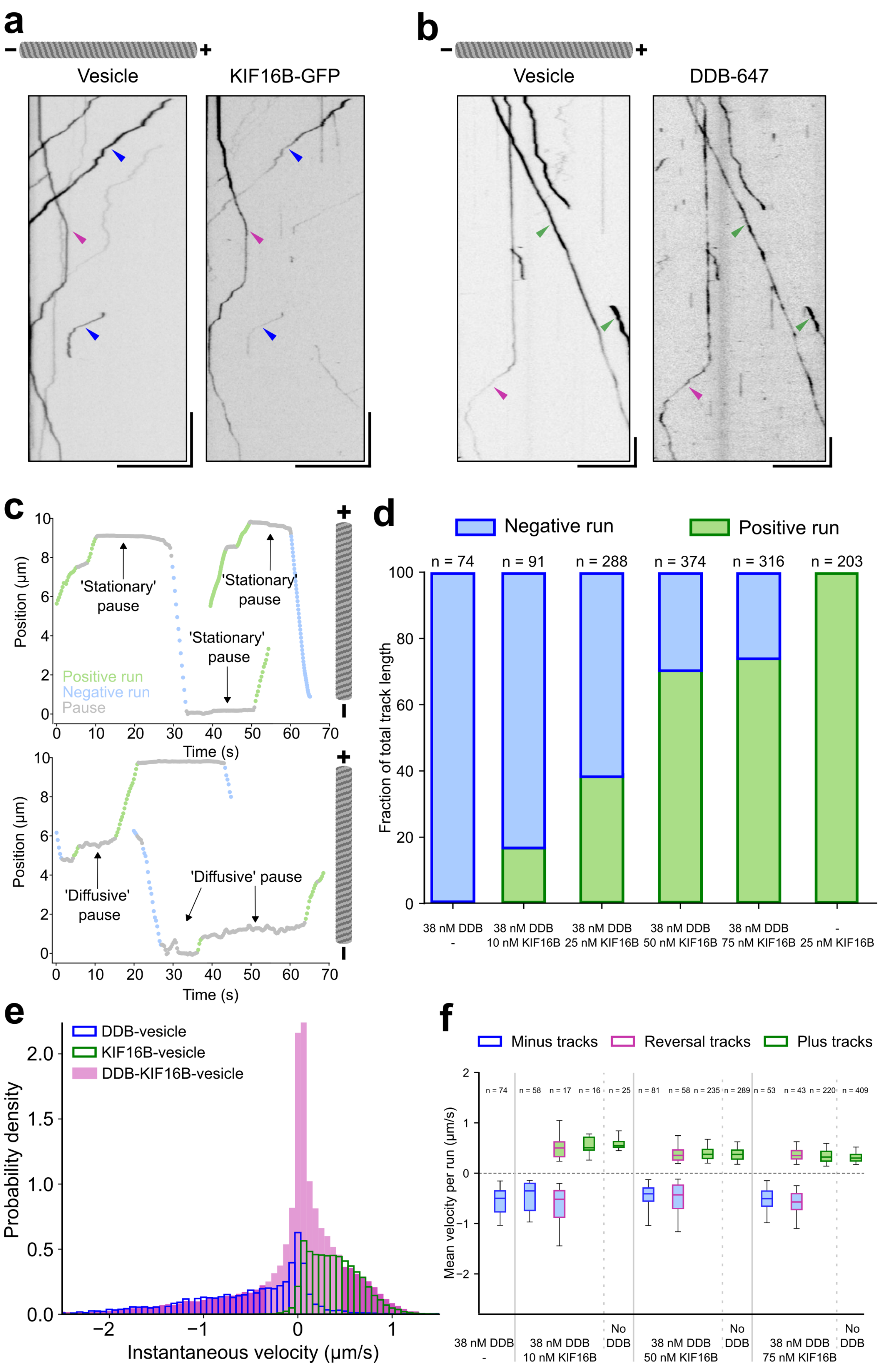
a
Vesicle
KIF16B-GFP
b
Vesicle
DDB-647
c
Position (µm)
Time (s)
'Stationary' pause
'Stationary' pause
'Stationary' pause
Positive run
Negative run
Pause
'Diffusive' pause
'Diffusive' pause
d
Negative run
Positive run
Fraction of total track length
n = 74
n = 91
n = 288
n = 374
n = 316
n = 203
38 nM DDB -
38 nM DDB 10 nM KIF16B
38 nM DDB 25 nM KIF16B
38 nM DDB 50 nM KIF16B
38 nM DDB 75 nM KIF16B
- 25 nM KIF16B
e
DDB-vesicle
KIF16B-vesicle
DDB-KIF16B-vesicle
Probability density
Instantaneous velocity (µm/s)
f
Minus tracks
Reversal tracks
Plus tracks
Mean velocity per run (µm/s)
n = 74
n = 58
n = 17
n = 16
n = 25
n = 81
n = 58
n = 235
n = 289
n = 53
n = 43
n = 220
n = 409
38 nM DDB -
38 nM DDB 10 nM KIF16B
No DDB
38 nM DDB 50 nM KIF16B
No DDB
38 nM DDB 75 nM KIF16B
No DDB

Supplementary Figure 3 | Opposing motors do not affect the velocity of the driving motors. a) Kymographs of Atto647N-labelled vesicles (left panel) incubated with unlabelled DDB and KIF16B-eGFP (right panel) showing colocalizations between KIF16B-eGFP and a reversing vesicle (magenta arrowheads) and two minus-end moving vesicles (blue arrowheads). Scale bars: vertical 20s, horizontal 23 µm. **b)** Kymographs of Atto488-labelled vesicles (left panel) incubated with Alexa647-labelled DDB (Dynein-647; right panel) and unlabelled KIF16B showing colocalizations between Dynein-647 and a reversing vesicle (magenta arrowhead) and two plus-end moving vesicles (green arrowheads). Scale bars: vertical 5s, horizontal 5 µm. **c)** Position-time tracks of DDB-KIF16B-vesicles with stationary pauses and diffusive pauses. **d)** Proportion of negative and positive runs obtained from DDB-KIF16B-vesicles incubated with 38 nM DDB and various concentrations of KIF16B (10, 25, 50, 75 nM). DDB-vesicles and KIF16B-vesicles were used as controls. n represents the total number of tracked vesicles for a given condition (data pooled from two independent experiments). **e)** Probability densities of instantaneous velocity histograms of DDB-vesicles (Fig. 2b) and KIF16B-vesicles (Fig. 2c) were uniformly scaled (factor of 0.355) and overlaid onto the instantaneous velocity histogram of DDB-KIF16B-vesicles (Fig. 2d). Note that the shape of the high velocity tails (dark magenta) matches between the dual- and single-motor vesicles. **f)** Comparative mean velocity boxplot of negative and positive runs obtained from dual-motor vesicles (38 nM DDB and 10, 50, 75 nM KIF16B) and single-motor vesicles. n represents the number of tracked vesicles. Box plots indicate median (middle line), 25th, 75th percentile (box) and 5th and 95th percentile (whiskers). Numerical values and statistical comparisons are presented in Supplementary Tables 2 and 3.

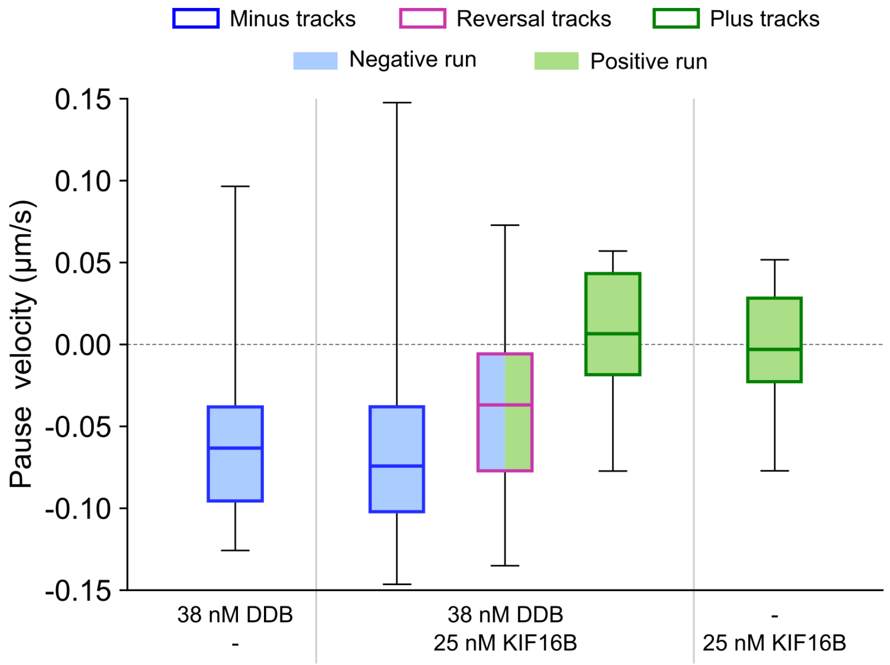

Supplementary Figure 4 | Segment velocities during vesicle pauses. Box plot of mean velocities during pauses of DDB-vesicles, KIF16B-vesicles and DDB-KIF16B-vesicles. The plots indicate median (middle line), 25th, 75th percentile (box) and 5th and 95th percentile (whiskers).

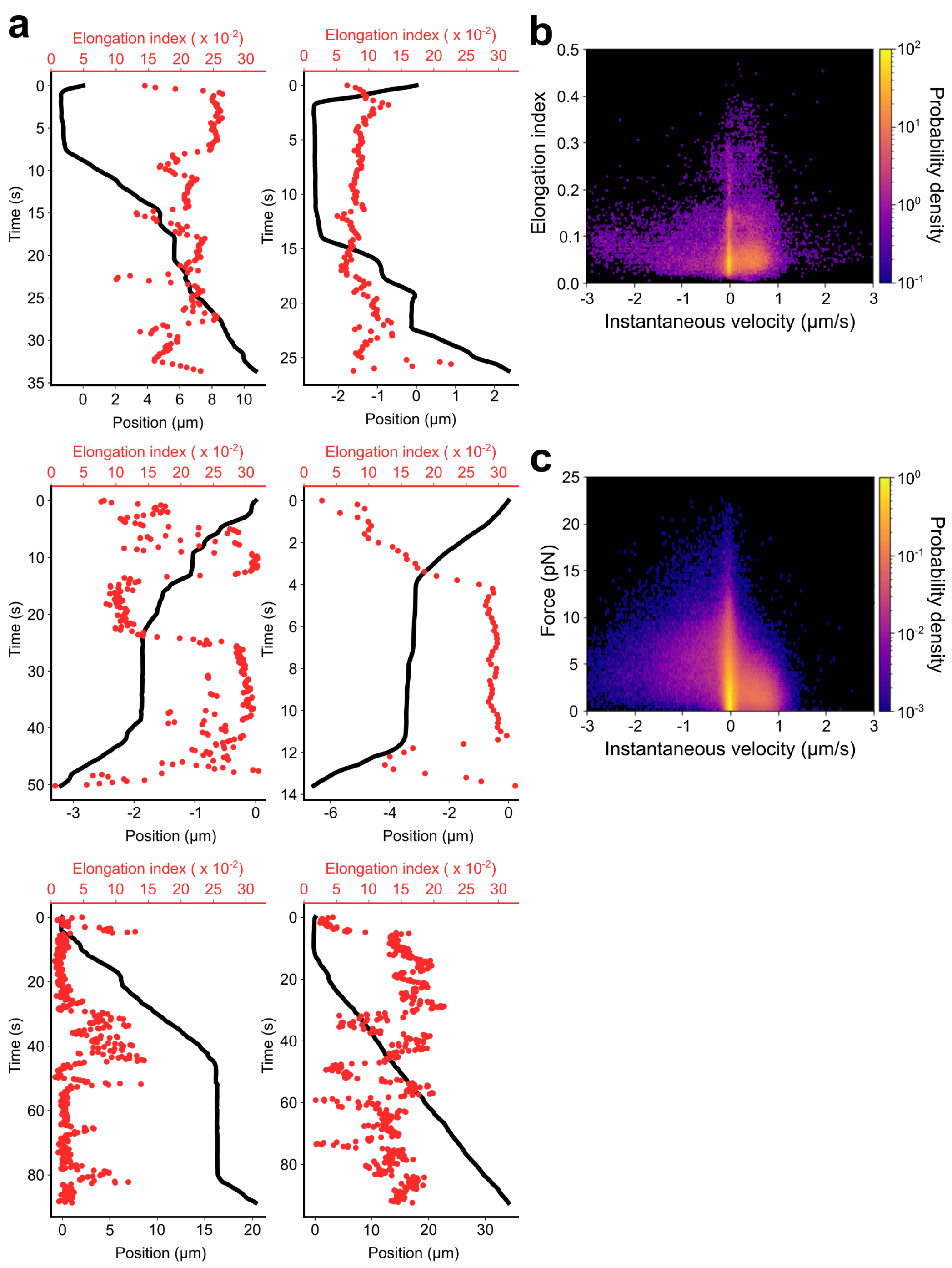

Supplementary Figure 5 | Vesicle elongations are dominant during pausing. a) Position (black) and elongation index (red) of reversing (upper panel), minus-end directed (middle panel) and plus-end directed (lower panel) dual-motor vesicles. Elongation index measures the shape of the vesicle and is calculated as the ratio of the difference to the sum of long and short axis of the vesicles obtained by fitting a stretched Gaussian to the vesicle image during particle tracking. Higher elongation indices indicate elongated vesicles. **b)** Heatmap of the probability density for elongation index and instantaneous velocity of dual-motor vesicles (from experimental data). High densities of higher elongation indices are observed at lower velocities. **c)** Heatmap of the probability density for total absolute force acting on a dual-motor cargo and instantaneous velocity (from simulated data). High densities of higher forces are observed at lower velocities.

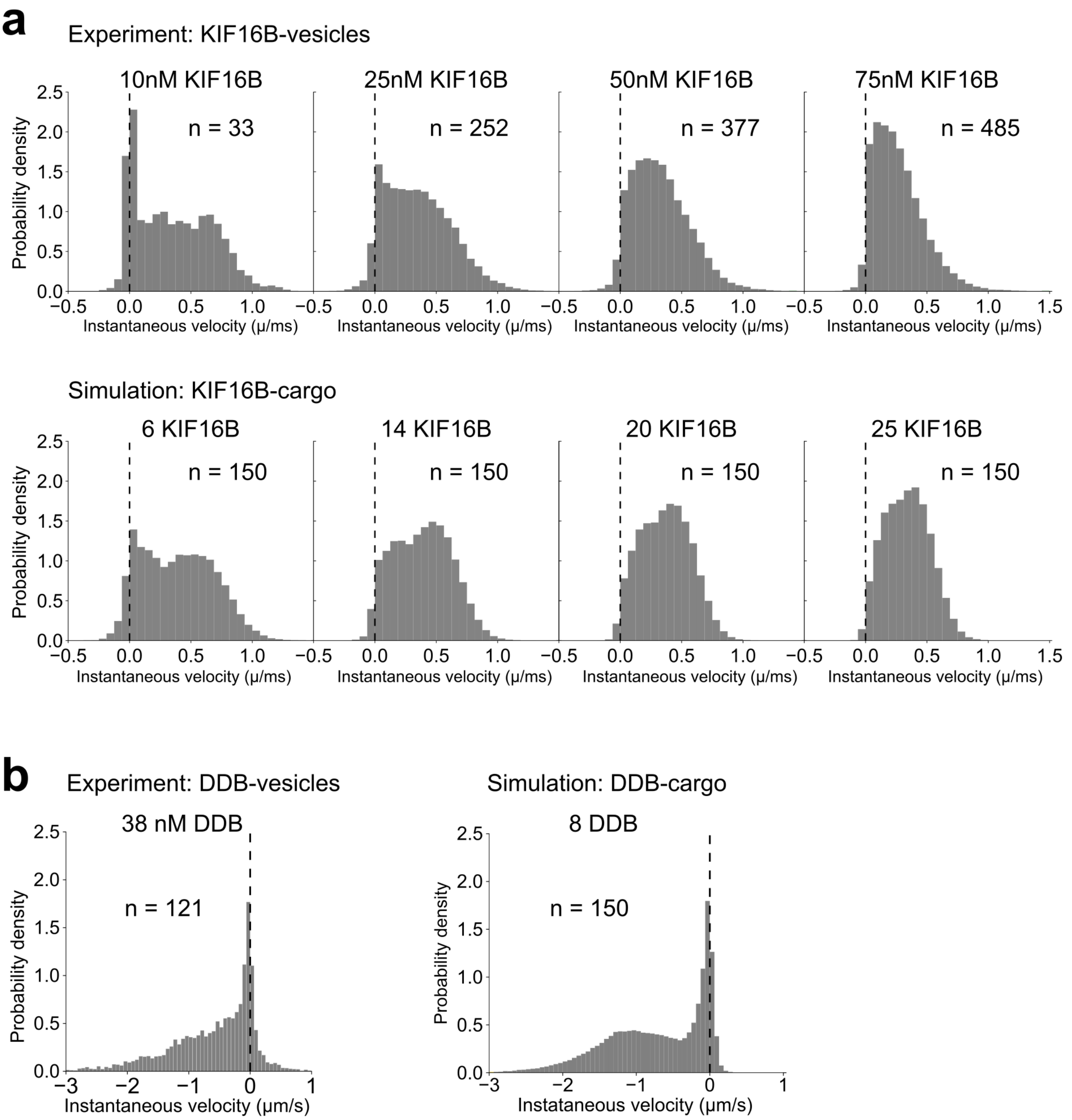

Supplementary Figure 6 | Stochastic numerical simulation can recapitulate instantaneous velocity profiles of single-motor vesicles. a) Instantaneous velocity histograms of vesicles incubated with 10, 25, 50 and 75 nM KIF16B (upper panel) and of cargoes simulated with 6, 14, 20 and 25 KIF16B motors in the attachment area (lower panel). **b)** Instantaneous velocity histograms of vesicles incubated with 38 nM DDB (left) and of cargoes simulated with 8 DDB motors in the attachment area (right). n represents the number of motor-bound vesicles/cargoes used to construct the histograms.

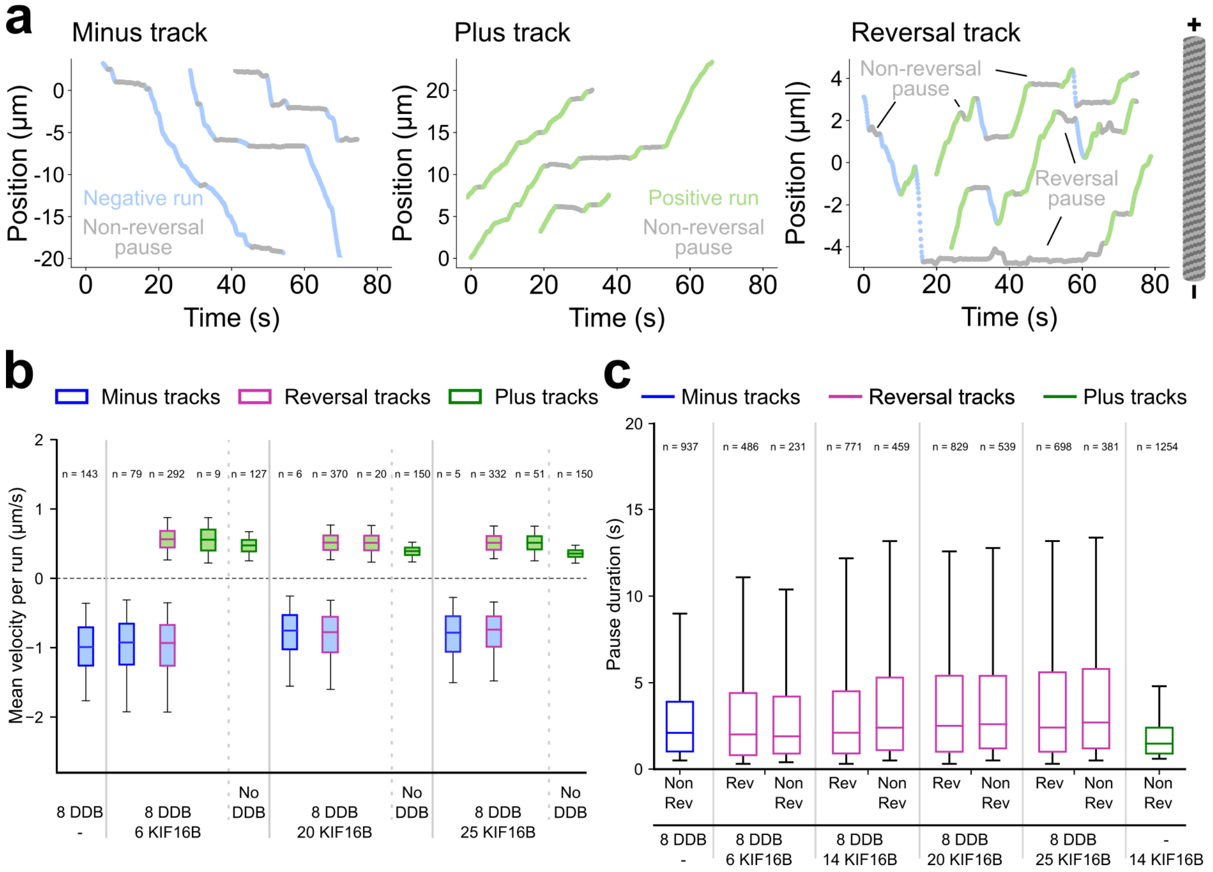

Supplementary Figure 7 | Simulated assemblies of dual-motor cargoes recapitulate the experimental characteristics of runs and pauses of dual-motor vesicles. a) Segmented minus tracks (left), plus tracks (middle), and reversal tracks (right) obtained from simulations of dual-motor cargoes (8 DDB and 14 KIF16B motors) and single-motor cargoes. **b)** Comparitive mean velocity box plot of negative and positive runs obtained from simulation of dual-motor (8 DDB and 6, 20, 25 KIF16B motors) and single motor cargoes. n represents the number of analyzed cargoes. Numerical values and statistical comparison are presented in Supplementary Tables 6 and 7. **c)** Comparitive pause duration box plots of reversal and non-reversal pauses obtained from simulation of dual-motor (8 DDB and 6, 14, 20, 25 KIF16B motors) and single-motor cargoes. n represents the number of pauses. Statistical comparisons are presented in Supplementary Table 8. Box plots indicate median (middle line), 25th, 75th percentile (box) and 5th and 95th percentile (whiskers).

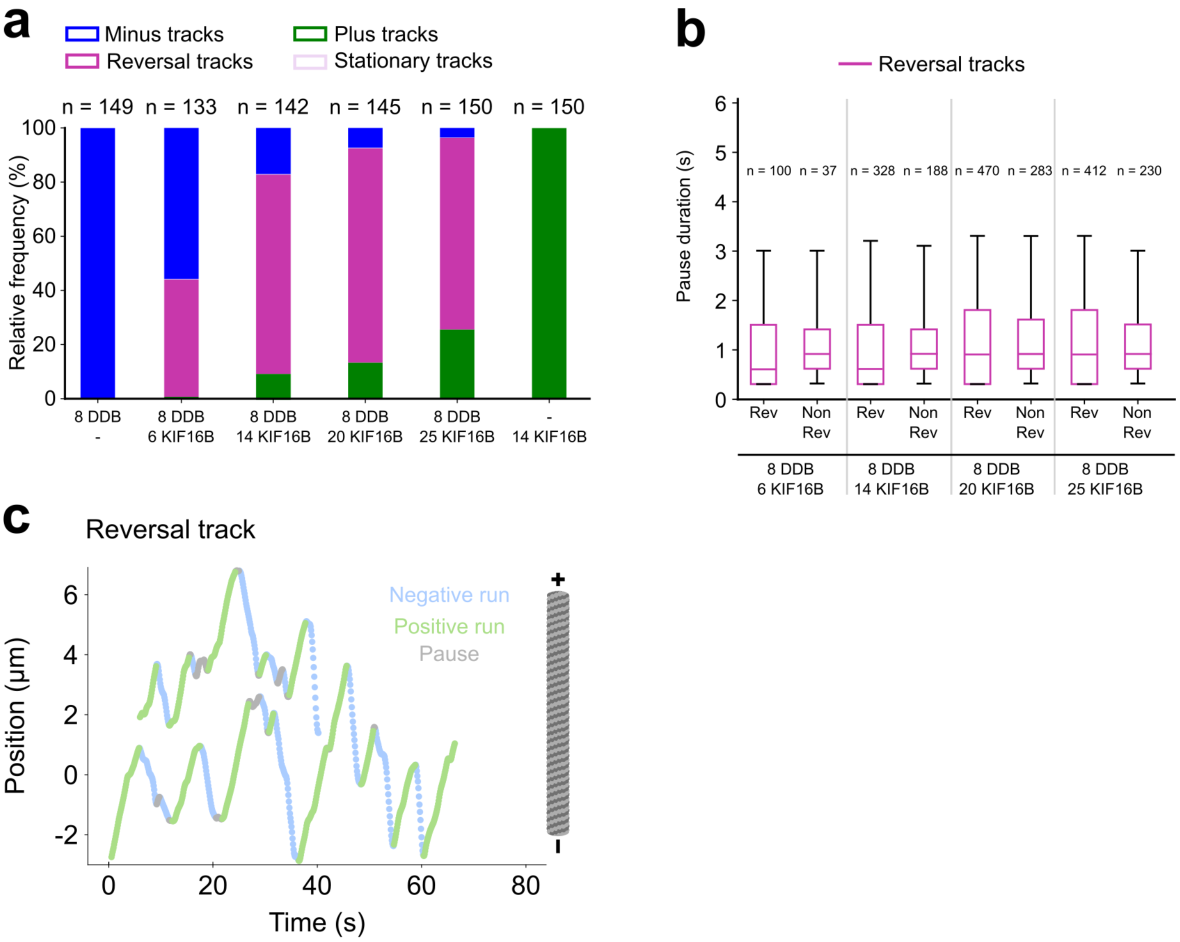

Supplementary Figure 8 | Inactive motors affect the dynamics and duration of pauses. **a)** Proportions of minus (blue), plus (green), reversal (magenta), and stationary (lilac) tracks obtained from the simulated data in the absence of inactive motors. Tracks are obtained from simulations of cargoes with 8 DDB motors and varying numbers of 6, 14, 20, 25 KIF16B motors. Simulations with either 8 DDB or 14 KIF16B motors only are shown as controls. n represents the number of pauses. **b)** Comparitive pause duration box plots of reversal (rev) and non-reversal (non-rev) pauses obtained from simulation of dual-motor and single-motor cargoes. Box plots indicate median (middle line), 25th, 75th percentile (box) and 5th and 95th percentile (whiskers). Statistical comparisons are presented in Supplementary Table 9. **c)** Representative traces of a dual-motor cargoes exhibiting directional reversals. The paused phases resemble a diffusive states.

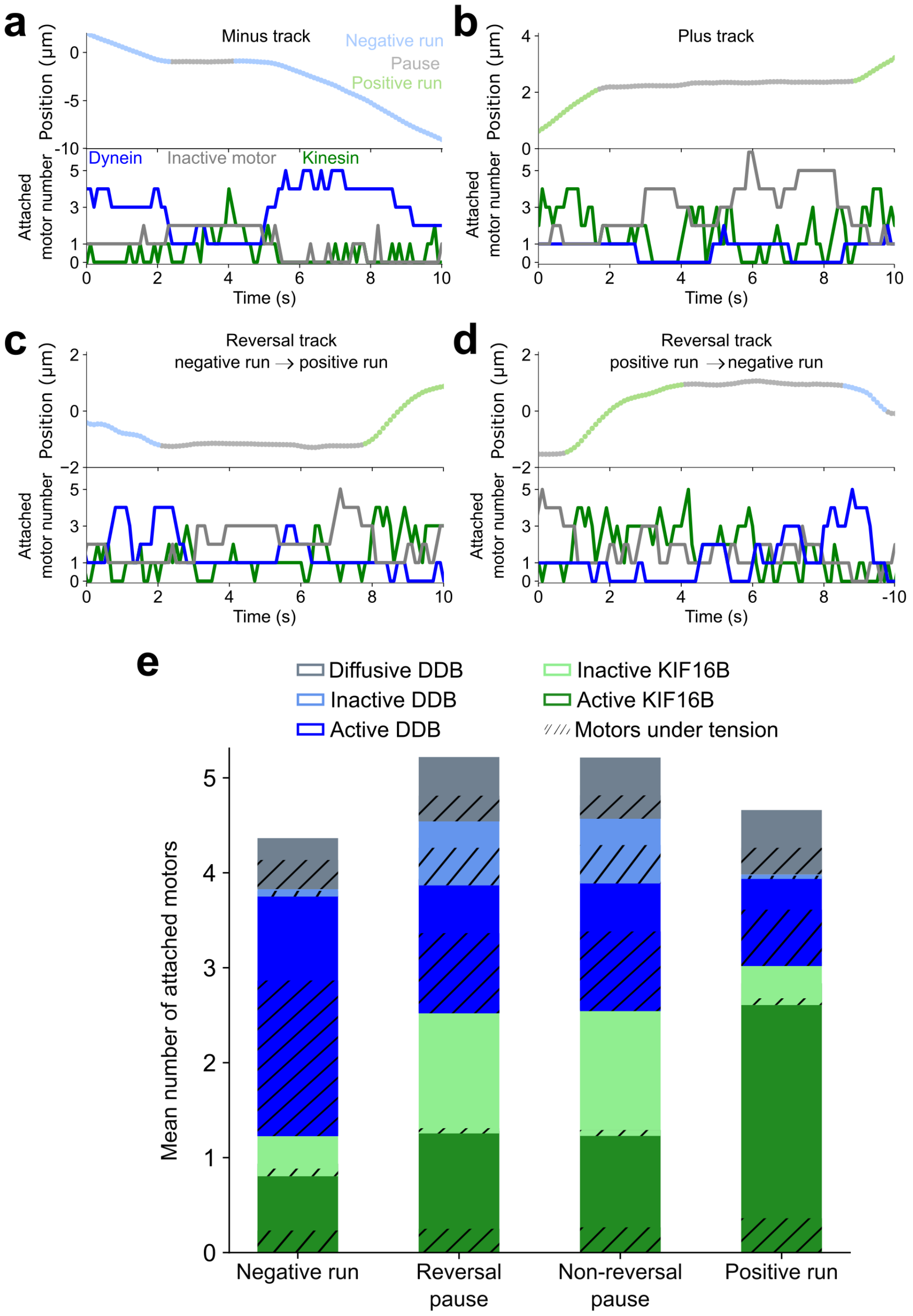

Supplementary Figure 9 | Number of motors bound to the microtubule during different phases of motility of a simulated cargo. **a – d)** Position-time plots of simulated dual-motor cargoes (8 DDB and 14 KIF16B motors) and the number of DDB (blue), KIF16B (green) and inactive (grey) motors attached to the microtubule at any given instant. Inactive motors include inactive KIF16B motors as well as inactive and diffusive DDB motors. **e)** Stacked bar plots of mean number of motors attached to the microtubule during negative runs, positive runs, reversal and non-reversal pauses of reversal tracks from simulation with 8 DDB and 14 KIF16B motors. Shaded areas show motors which are under tension.

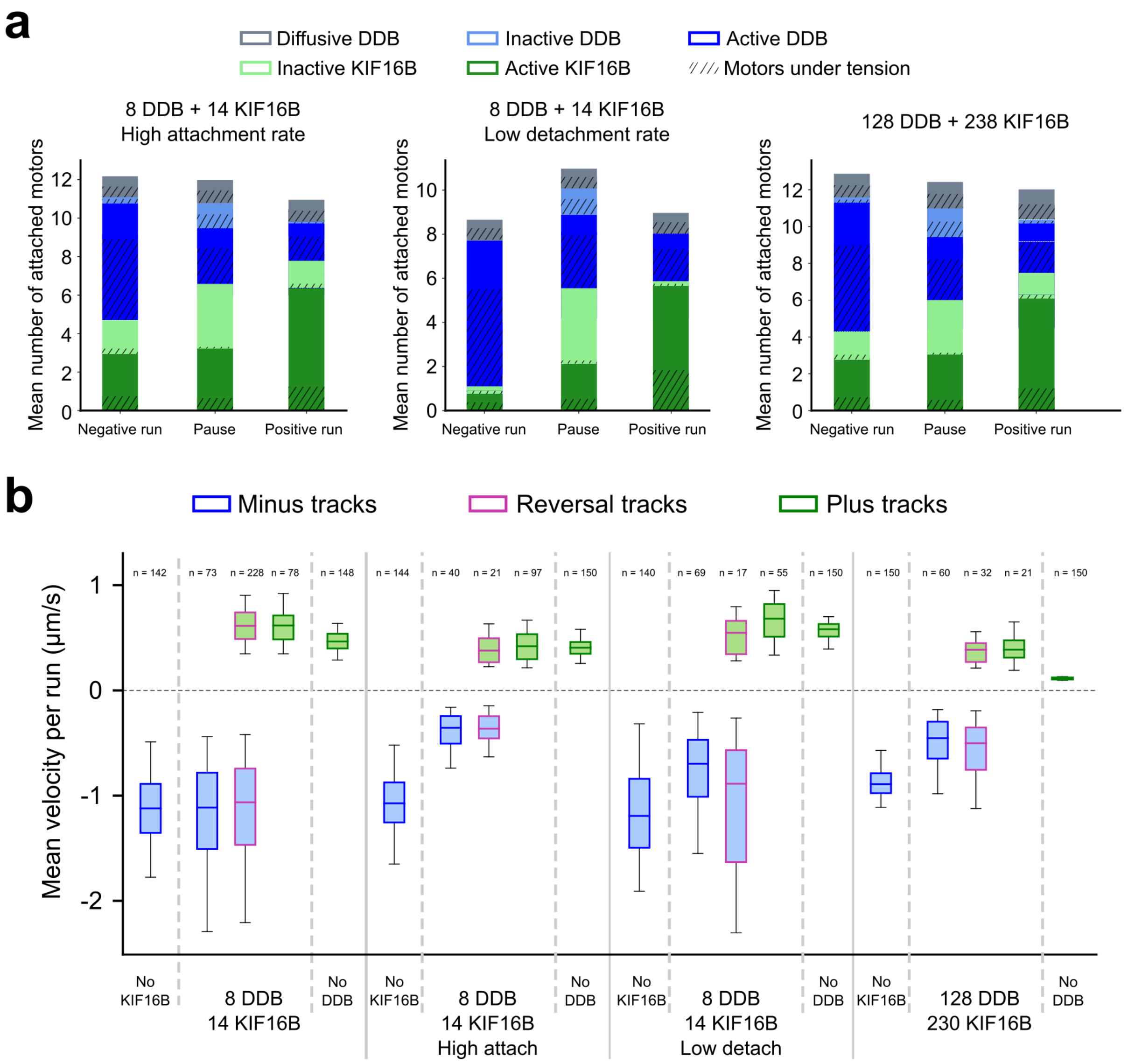

Supplementary Figure 10 | Increasing the number of attached motors affects the characteristics of dual-motor cargo transport. a) Stacked bar histograms of mean number of motors attached to the microtubule during negative runs, positive runs and paused phases when dual-motor cargoes were simulated with higher attachment rate motors (left, 20 s^{-1} vs 0.625 s^{-1} for DDB, 40 s^{-1} vs 1.25 s^{-1} for KIF16B), lower detachment rate motors (middle, 0.022 s^{-1} vs 0.44 s^{-1} for active DDB, 0.009 s^{-1} vs 0.18 s^{-1} for inactive and diffusive DDB, 0.0635 s^{-1} vs 1.27 s^{-1} for active KIF16B, 0.035 s^{-1} vs 0.7 s^{-1} for inactive KIF16B) and higher number of motors (right, 128 DDB and 230 KIF16B). Shaded areas show motors which are under tension. **b)** Comparitive mean velocity box plots of negative and positive runs of dual-motor cargoes simulated with altered motor attachment/detachment kinetics and numbers. Box plots indicate median (middle line), 25th, 75th percentile (box) and 5th and 95th percentile (whiskers). n represents the number of analyzed cargoes. Numerical values and statistical comparisons are presented in Supplementary Tables 10 and 11, respectively.

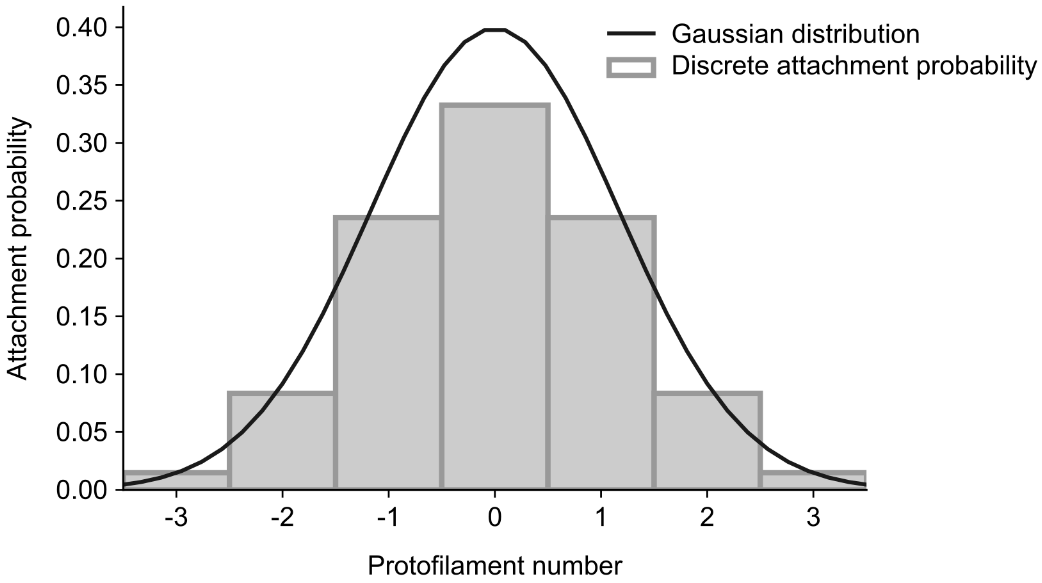

Supplementary Figure 11 | Gaussian distributed attachment to protofilaments. Bar plot shows the attachment probability of either motor type distributed over the microtubule protofilaments. Attachment probability is the highest for the central protofilament (number zero) and reduces in a Gaussian manner for protofilaments away from the central protofilament. The corresponding Gaussian distribution (mean zero and standard deviation one) is shown as solid line. 7 out of 14 protofilaments of a microtubule are accessible to the motors.

Supplementary Tables

Mol (%)	Short-form	Proper name	Function
74	DOPC	1,2-dioleoyl-sn-glycero-3-phosphocholine	Structural phospholipid
20	DOPE	1,2-dioleoyl-sn-glycero-3-phosphoethanolamine	Enhance KIF16B binding
3	PI(3)P	1,2-dioleoyl-sn-glycero-3-phospho-(1'-myo-inositol-3'-phosphate)	KIF16B binding
3	DGS- NTA(Ni)	1,2-dioleoyl-sn-glycero-3-[(N-(5-amino-1-carboxypentyl)iminodiacetic acid)succinyl] (nickel salt)	DDB binding
0.01	DOPE-Atto647N	1,2-dioleoyl-sn-glycero-3-phosphoethanolamine labelled with Atto647N	Fluorescent marker (for all data except Fig. S3b)
		or	
0.1	DOPE-Atto488	1,2-dioleoyl-sn-glycero-3-phosphoethanolamine labelled with Atto488	Fluorescent marker (for data in Fig. S3b)

Supplementary Table 1 | Phospholipid composition of vesicles. Phospholipids were mixed in the indicated molar ratios in chloroform, dried first under a stream of nitrogen gas and then in vacuum for 3-4 hours. Multilamellar vesicles (MLVs) were synthesized by rehydrating the lipid film in dynein buffer B (25 mM HEPES pH 7.4, 50 mM potassium acetate, 2 mM magnesium acetate) supplemented with 5 % (w/v) sucrose for 30 min at 37 °C (with periodic agitation). An aliquot of MLVs (total lipid mass of 50 µg) was used to synthesize large unilamellar vesicles (LUVs) as outlined in the main text (see also Materials and Methods).

Motor composition	Direction	Velocity (μm s^{-1}) (median ± IQR)	Number of vesicles
38 nM DDB	Minus	-0.56 ± 0.49	74
38 nM DDB + 10 nM KIF16B	Minus	-0.45 ± 1.49	58
	Reversal-Minus	-0.65 ± 0.56	17
	Reversal-Plus	0.50 ± 0.34	17
	Plus	0.46 ± 0.21	16
10 nM KIF16B	Plus	0.60 ± 0.13	25
38 nM DDB + 25 nM KIF16B	Minus	-0.58 ± 0.51	120
	Reversal-Minus	-0.69 ± 0.46	87
	Reversal-Plus	0.47 ± 0.26	87
	Plus	0.41 ± 0.28	81
25 nM KIF16B	Plus	0.39 ± 0.25	203
38 nM DDB + 50 nM KIF16B	Minus	-0.46 ± 0.35	81
	Reversal-Minus	-0.63 ± 0.41	58
	Reversal-Plus	0.40 ± 0.23	58
	Plus	0.39 ± 0.22	235
50 nM KIF16B	Plus	0.39 ± 0.23	289
38 nM DDB + 75 nM KIF16B	Minus	-0.52 ± 0.30	53
	Reversal-Minus	-0.59 ± 0.39	43
	Reversal-Plus	0.38 ± 0.31	43
	Plus	0.34 ± 0.23	220
75 nM KIF16B	Plus	0.31 ± 0.18	409

Supplementary Table 2 | Median ± IQR of mean instantaneous velocities of experimental vesicles. Velocities of minus-end directed runs (blue background) and plus-end directed runs (green background) of single- and dual-motor vesicles.

Motor composition and test	p-values with Bonferroni correction (3 comparisons) - Mean velocity of negative runs-			
38 nM DDB + 10 nM KIF16B **Two sample t-test**		**Only DDB**	**Minus**	**Reversal-Minus**
	**Only DDB**	1.000	0.288	1.000
	**Minus**	0.288	1.000	0.142
	**Reversal-Minus**	1.000	0.142	1.000
38 nM DDB + 25 nM KIF16B **Two sample t-test**		**Only DDB**	**Minus**	**Reversal-Minus**
	**Only DDB**	1.000	1.000	0.348
	**Minus**	1.000	1.000	0.341
	**Reversal-Minus**	0.348	0.341	1.000
38 nM DDB + 50 nM KIF16B **Two sample t-test**		**Only DDB**	**Minus**	**Reversal-Minus**
	**Only DDB**	1.000	0.364	1.000
	**Minus**	0.364	1.000	0.543
	**Reversal-Minus**	1.000	0.543	1.000
38 nM DDB + 75 nM KIF16B **Two sample t-test**		**Only DDB**	**Minus**	**Reversal-Minus**
	**Only DDB**	1.000	0.802	1.000
	**Minus**	0.802	1.000	0.817
	**Reversal-Minus**	1.000	0.817	1.000

Motor composition and test	p-values with Bonferroni correction (3 comparisons) - Mean velocity of positive runs-			
38 nM DDB + 10 nM KIF16B **Welch test**		**Reversal-Plus**	**Plus**	**Only KIF16B**
	**Reversal-Plus**	1.000	0.727	1.000
	**Plus**	0.727	1.000	0.152
	**Only KIF16B**	1.000	0.152	1.000
38 nM DDB + 25 nM KIF16B **Two sample t-test**		**Reversal-Plus**	**Plus**	**Only KIF16B**
	**Reversal-Plus**	1.000	0.416	0.001
	**Plus**	0.416	1.000	0.840
	**Only KIF16B**	0.001	0.840	1.000
38 nM DDB + 50 nM KIF16B **Mann Whitney U-test**		**Reversal-Plus**	**Plus**	**Only KIF16B**
	**Reversal-Plus**	1.000	1.000	1.000
	**Plus**	1.000	1.000	1.000
	**Only KIF16B**	1.000	1.000	1.000
38 nM DDB + 75 nM KIF16B **Mann Whitney U-test**		**Reversal-Plus**	**Plus**	**Only KIF16B**
	**Reversal-Plus**	1.000	0.645	0.013
	**Plus**	0.645	1.000	0.011
	**Only KIF16B**	0.013	0.011	1.000

Supplementary Table 3 | Statistical comparisons of experimental negative and positive velocities of all dual-motor vesicles. Supplementary to Fig. 3c and Supplementary Fig. S3f. All tests were two-tailed.

Parameter	Value	Discussion and references
Attachment rate	2.5 s^{-1} (0.625 s^{-1})	Standard (in the presence of KIF16B)[1,2]
Force-free detachment rate, active motors	0.44 s^{-1}	Obtained from experiments (**Fig. 1** and **Supplementary Fig. 1**)
Force-free detachment rate, inactive motors	0.18 s^{-1}	Obtained from comparing simulated and experimental DDB-vesicle instantaneous velocities.
Stall force	4 pN	Ref[3]
Detachment force (exponential)	3 pN	Ref[4]
Force-free velocity at 2.5 mM ATP	Instantaneous velocity distribution of single DDB (ignoring positive velocities)	Experiment (**Fig. 1)**
Backward velocity	6 nm s^{-1}	Same order of magnitude as [5]
Diffusion rate of diffusive DDB motors per 8 nm	250 s^{-1}	Ref[6]
Stiffness	0.065 pN.nm^{-1}	Ref[7]
Untensioned length	30 nm	Same order of magnitude as [8]
Motor radius on the microtubule	24 nm	Approximated from EM images of Ref[9,10]

Supplementary Table 4 | DDB parameters used in numerical simulations

Parameter	Value	Discussion and references
Attachment rate	5 s^{-1} (1.25 s^{-1})	Standard (in the presence of DDB)[1,2]
Force-free detachment rate	1.27 s^{-1}	Obtained from experiments (**Fig. 1** and **Supplementary Fig. 1**).
Force-free detachment rate for inactive motors	0.70 s^{-1}	Obtained from comparing simulated and experimental KIF16B-vesicle instantaneous velocities.
Detachment force	2 pN	Same order of magnitude as [11]
Stall force	6 pN	Ref[11]
Force-free velocity at 2.5 mM ATP	Instantaneous velocity distribution of single KIF16B motors (ignoring negative velocities)	Experiment (**Fig. 1)**
Backward velocity	6 nm s^{-1}	Same order of magnitude as [5]
Stiffness	0.3 pN.nm^{-1}	Ref[7]
Untensioned length	70 nm	Same order of magnitude as [12,13]
Motor radius on the microtubule	4 nm	Same order of magnitude as [14]

Supplementary Table 5 | KIF16B parameters used in numerical simulations

Motor composition	Direction	Velocity (μm s^{-1}) (median ± IQR)	Number of cargoes
8 DDB	Minus	-0.98 ± 0.55	143
8 DDB + 6 KIF16B	Minus	-0.92 ± 0.59	79
	Reversal-Minus	-0.93 ± 0.58	292
	Reversal-Plus	0.56 ± 0.24	292
	Plus	0.56 ± 0.30	9
6 KIF16B	Plus	0.47 ± 0.16	127
8 DDB + 14 KIF16B	Minus	-0.82 ± 0.51	13
	Reversal-Minus	-0.83 ± 0.52	351
	Reversal-Plus	0.53 ± 0.20	351
	Plus	0.52 ± 0.25	19
14 KIF16B	Plus	0.43 ± 0.13	148
8 DDB + 20 KIF16B	Minus	-0.75 ± 0.49	6
	Reversal-Minus	-0.77 ± 0.51	370
	Reversal-Plus	0.51 ± 0.21	370
	Plus	0.51 ± 0.21	20
20 KIF16B	Plus	0.39 ± 0.11	150
8 DDB + 25 KIF16B	Minus	-0.77 ± 0.51	5
	Reversal-Minus	-0.73 ± 0.44	332
	Reversal-Plus	0.51 ± 0.20	332
	Plus	0.51 ± 0.19	51
25 KIF16B	Plus	0.36 ± 0.10	150

Supplementary Table 6 | Median ± IQR of means of instantaneous velocities per run of simulated cargoes. Velocities of minus-end directed runs (blue background) and plus-end directed runs (green background) of simulated single- and dual-motor cargoes. Supplementary to Fig. 5c and Supplementary Fig. S7b. Means are weighted and produced with resampled tracks (see model description below).

Motor composition and test	p-values with Bonferroni correction (3 comparisons) - Mean velocity of negative runs-			
8 DDB + 6 KIF16B **Weighted Kolmogorov-Smirnov test**		**Only DDB**	**Minus**	**Reversal-Minus**
	**Only DDB**	1.000	0.000	10^{-194}
	**Minus**	0.000	1.000	10^{-30}
	**Reversal-Minus**	10^{-194}	10^{-30}	1.000
8 DDB + 14 KIF16B **Weighted Kolmogorov-Smirnov test**		**Only DDB**	**Minus**	**Reversal-Minus**
	**Only DDB**	1.000	0.000	0.000
	**Minus**	0.000	1.000	10^{-221}
	**Reversal-Minus**	0.000	10^{-221}	1.000
8 DDB + 20 KIF16B **Weighted Kolmogorov-Smirnov test**		**Only DDB**	**Minus**	**Reversal-Minus**
	**Only DDB**	1.000	0.000	0.000
	**Minus**	0.000	1.000	10^{-266}
	**Reversal-Minus**	0.000	10^{-266}	1.000
8 DDB + 25 KIF16B **Weighted Kolmogorov-Smirnov test**		**Only DDB**	**Minus**	**Reversal-Minus**
	**Only DDB**	1.000	0.000	0.000
	**Minus**	0.000	1.000	0.000
	**Reversal-Minus**	0.000	0.000	1.000

Motor composition and test	p-values with Bonferroni correction (3 comparisons) - Mean velocity of positive runs-			
8 DDB + 6 KIF16B **Weighted Kolmogorov-Smirnov test**		**Reversal-Plus**	**Plus**	**Only KIF16B**
	**Reversal-Plus**	1.000	10^{-87}	0.000
	**Plus**	10^{-87}	1.000	0.000
	**Only KIF16B**	0.000	0.000	1.000
8 DDB + 14 KIF16B **Weighted Kolmogorov-Smirnov test**		**Reversal-Plus**	**Plus**	**Only KIF16B**
	**Reversal-Plus**	1.000	10^{-309}	0.000
	**Plus**	10^{-309}	1.000	0.000
	**Only KIF16B**	0	0.000	1.000
8 DDB + 20 KIF16B **Weighted Kolmogorov-Smirnov test**		**Reversal-Plus**	**Plus**	**Only KIF16B**
	**Reversal-Plus**	1.000	10^{-120}	0.000
	**Plus**	10^{-120}	1.000	0.000
	**Only KIF16B**	0.000	0.000	1.000
8 DDB + 25 KIF16B **Weighted Kolmogorov-Smirnov test**		**Reversal-Plus**	**Plus**	**Only KIF16B**
	**Reversal-Plus**	1.000	10^{-106}	0.000
	**Plus**	10^{-106}	1.000	0.000
	**Only KIF16B**	0.000	0.000	1.000

Supplementary Table 7 | Statistical comparisons of simulated negative and positive velocities of all dual-motor cargoes. Supplementary to Fig. 5c and Supplementary Fig. S7b. All tests were two-tailed.

Motor composition and test	p-values with Bonferroni correction (3 comparisons)				
8 DDB + 6 KIF16B **Weighted Kolmogorov-Smirnov test**		**Only DDB**	**Reversal pause**	**Non-Reversal pause**	**Only KIF16B**
	**Only DDB**	1.000	10^{-273}	10^{-204}	0
	**Reversal pause**	10^{-273}	1.000	10^{-187}	0
	**Non-Reversal pause**	10^{-204}	10^{-187}	1.000	0
	**Only KIF16B**	0	0	0	1.000
8 DDB + 14 KIF16B **Weighted Kolmogorov-Smirnov test**		**Only DDB**	**Reversal pause**	**Non-Reversal pause**	**Only KIF16B**
	**Only DDB**	1.000	0	0	0
	**Reversal pause**	0	1.000	0	0
	**Non-Reversal pause**	0	0	1.000	0
	**Only KIF16B**	0	0	0	1.000
8 DDB + 20 KIF16B **Weighted Kolmogorov-Smirnov test**		**Only DDB**	**Reversal pause**	**Non-Reversal pause**	**Only KIF16B**
	**Only DDB**	1.000	0	0	0
	**Reversal pause**	0	1.000	0	0
	**Non-Reversal pause**	0	0	1.000	0
	**Only KIF16B**	0	0	0	1.000
8 DDB + 25 KIF16B **Weighted Kolmogorov-Smirnov test**		**Only DDB**	**Reversal pause**	**Non-Reversal pause**	**Only KIF16B**
	**Only DDB**	1.000	0	0	0
	**Reversal pause**	0	1.000	0	0
	**Non-Reversal pause**	0	0	1.000	0
	**Only KIF16B**	0	0	0	1.000

Supplementary Table 8 | Statistical comparisons of simulated reversal and non-reversal pause duration of reversal tracks for all dual-motor cargoes. Supplementary to Supplementary Fig. S7c. All tests were two-tailed.

Motor composition and test	p-values		
8 DDB + 6 KIF16B **Weighted Kolmogorov-Smirnov test**		**Reversal pause**	**Non-Reversal pause**
	**Reversal pause**	1.000	0.000
	**Non-Reversal pause**	0.000	1.000
8 DDB + 14 KIF16B **Weighted Kolmogorov-Smirnov test**		**Reversal pause**	**Non-Reversal pause**
	**Reversal pause**	1.000	0.000
	**Non-Reversal pause**	0.000	1.000
8 DDB + 20 KIF16B **Weighted Kolmogorov-Smirnov test**		**Reversal pause**	**Non-Reversal pause**
	**Reversal pause**	1.000	0.000
	**Non-Reversal pause**	0.000	1.000
8 DDB + 25 KIF16B **Weighted Kolmogorov-Smirnov test**		**Reversal pause**	**Non-Reversal pause**
	**Reversal pause**	1.000	0.000
	**Non-Reversal pause**	0.000	1.000

Supplementary Table 9 | Statistical comparisons of reversal and non-reversal pause duration of reversal tracks for all simulated dual-motor cargoes without inactive motors. Supplementary to Supplementary Fig. S8b. All tests were two-tailed.

Condition	Motor composition	Direction	Velocity (μm s^{-1}) (median ± IQR)	Number of cargoes
Conventional attachment rate	8 DDB	Minus	-0.98 ± 0.55	143
	8 DDB + 14 KIF16B	Minus	-0.82 ± 0.51	13
		Reversal-Minus	-0.83 ± 0.52	351
		Reversal-Plus	0.53 ± 0.20	351
		Plus	0.52 ± 0.25	19
	14 KIF16B	Plus	0.43 ± 0.13	148
High attachment rate	8 DDB	Minus	-0.92 ± 0.39	143
	8 DDB + 14 KIF16B	Minus	-0.33 ± 0.21	39
		Reversal-Minus	-0.32 ± 0.25	17
		Reversal-Plus	0.40 ± 0.23	17
		Plus	0.38 ± 0.18	86
	14 KIF16B	Plus	0.38 ± 0.11	150
Low detachment rate	8 DDB	Minus	-1.05 ± 0.61	142
	8 DDB + 14 KIF16B	Minus	-0.68 ± 0.44	62
		Reversal-Minus	-0.77 ± 0.54	30
		Reversal-Plus	0.53 ± 0.32	30
		Plus	0.63 ± 0.31	52
	14 KIF16B	Plus	0.57 ± 0.12	150
High motor numbers	128 DDB	Minus	-0.83 ± 0.21	150
	128 DDB + 230 KIF16B	Minus	-0.42 ± 0.22	56
		Reversal-Minus	-0.42 ± 0.26	41
		Reversal-Plus	0.35 ± 0.15	41
		Plus	0.33 ± 0.19	15
	230 KIF16B	Plus	0.12 ± 0.02	150

Supplementary Table 10 | Median ± IQR of mean instantaneous velocities. Values are given for minus-end directed runs (blue background) and plus-end directed runs (green background) of simulated single- and dual-motor cargoes with conventional parameter set, high attachment rates, low detachment rates and higher number of motors. Supplementary to Supplementary Fig. S10b. Means are weighted and produced with resampled tracks (see model description below).

Motor composition and test	p-values with Bonferroni correction (3 comparisons) - Mean velocities of negative runs-			
8 DDB + 14 KIF16B High attachment rate **Weighted Kolmogorov-Smirnov test**		**Only DDB**	**Minus**	**Reversal-Minus**
	**Only DDB**	1.000	0.000	0.000
	**Minus**	0.000	1.000	10^{-34}
	**Reversal-Minus**	0.000	10^{-34}	1.000
8 DDB + 14 KIF16B Low detachment rate **Weighted Kolmogorov-Smirnov test**		**Only DDB**	**Minus**	**Reversal-Minus**
	**Only DDB**	1.000	0.000	0.000
	**Minus**	0.000	1.000	10^{-146}
	**Reversal-Minus**	0.000	10^{-146}	1.000
128 DDB + 230 KIF16B **Weighted Kolmogorov-Smirnov test**		**Only DDB**	**Minus**	**Reversal-Minus**
	**Only DDB**	1.000	0.000	0.000
	**Minus**	0.000	1.000	10^{-69}
	**Reversal-Minus**	0.000	10^{-69}	1.000
**Motor composition and test**	**p-values with Bonferroni correction (3 comparisons) - Mean velocities of positive runs-**			
8 DDB + 14 KIF16B High attachment rate **Weighted Kolmogorov-Smirnov test**		**Reversal-Plus**	**Plus**	**Only KIF16B**
	**Reversal-Plus**	1.000	10^{-29}	10^{-48}
	**Plus**	10^{-29}	1.000	0.000
	**Only KIF16B**	10^{-48}	0.000	1.000
8 DDB + 14 KIF16B Low detachment rate **Weighted Kolmogorov-Smirnov test**		**Reversal-Plus**	**Plus**	**Only KIF16B**
	**Reversal-Plus**	1.000	10^{-266}	10^{-250}
	**Plus**	10^{-266}	1.000	0.000
	**Only KIF16B**	10^{-250}	0.000	1.000
128 DDB + 230 KIF16B **Weighted Kolmogorov-Smirnov test**		**Reversal-Plus**	**Plus**	**Only KIF16B**
	**Reversal-Plus**	1.000	10^{-78}	0.000
	**Plus**	10^{-78}	1.000	0.000
	**Only KIF16B**	0.000	0.000	1.000

Supplementary Table 11 | Statistical comparisons of negative and positive velocities of all simulated dual-motor cargoes with high attachment rate, low detachment rate and higher number of motors. Supplementary to Supplementary Fig. S10b. All tests were two-tailed.

References

1. Leduc, C. *et al.* Cooperative extraction of membrane nanotubes by molecular motors. *Proc. Natl. Acad. Sci.* **101**, 17096–17101 (2004).
2. Müller, M. J. I., Klumpp, S. & Lipowsky, R. Tug-of-war as a cooperative mechanism for bidirectional cargo transport by molecular motors. *Proc. Natl. Acad. Sci.* **105**, 4609–4614 (2008).
3. Belyy, V. *et al.* The mammalian dynein–dynactin complex is a strong opponent to kinesin in a tug-of-war competition. *Nat. Cell Biol.* **18**, 1018–1024 (2016).
4. Ma, T.-C., Gicking, A. M., Feng, Q. & Hancock, W. O. Simulations suggest robust microtubule attachment of kinesin and dynein in antagonistic pairs. *Biophys. J.* **122**, 3299-3313 (2023).
5. Carter, N. J. & Cross, R. A. Mechanics of the kinesin step. *Nature* **435**, 308–312 (2005).
6. Feng, Q., Gicking, A. M. & Hancock, W. O. Dynactin p150 promotes processive motility of DDB complexes by minimizing diffusional behavior of dynein. *Mol. Biol. Cell* **31**, 782–792 (2020).
7. Ohashi, K. G. *et al.* Load-dependent detachment kinetics plays a key role in bidirectional cargo transport by kinesin and dynein. *Traffic* **20**, 284–294 (2019).
8. Monzon, G. A. *et al.* Stable tug-of-war between kinesin-1 and cytoplasmic dynein upon different ATP and roadblock concentrations. *J. Cell Sci.* **133**, jcs249938 (2020).
9. Schlager, M. A., Hoang, H. T., Urnavicius, L., Bullock, S. L. & Carter, A. P. In vitro reconstitution of a highly processive recombinant human dynein complex. *EMBO J.* **33**, 1855–1868 (2014).
10. Torisawa, T. *et al.* Autoinhibition and cooperative activation mechanisms of cytoplasmic dynein. *Nat. Cell Biol.* **16**, 1118–1124 (2014).
11. Gicking, A. M. *et al.* Kinesin-1, -2 and -3 motors use family-specific mechanochemical strategies to effectively compete with dynein during bidirectional transport. *eLife* **11**, e82228 (2022).
12. Hirokawa, N. *et al.* Submolecular domains of bovine brain kinesin identified by electron microscopy and monoclonal antibody decoration. *Cell* **56**, 867–878 (1989).
13. Kerssemakers, J., Howard, J., Hess, H. & Diez, S. The distance that kinesin-1 holds its cargo from the microtubule surface measured by fluorescence interference contrast microscopy. *Proc. Natl. Acad. Sci. U. S. A.* **103**, 15812–15817 (2006).
14. Sozański, K. *et al.* Small crowders slow down Kinesin-1 stepping by hindering motor domain diffusion. *Phys. Rev. Lett.* **115**, 218102 (2015).
