# Supplementary material for: Vesicles driven by dynein and kinesin exhibit directional reversals without regulators
Source: Nat Commun. 2023 Nov 20;14:7532. doi: 10.1038/s41467-023-42605-8 (PMC10662051; doi:10.1038/s41467-023-42605-8)
Supplement: Supplementary file 3 — Description of Additional Supplementary Files [file 41467_2023_42605_MOESM3_ESM.pdf]

Description of Additional Supplementary Files

File name: Supplementary Movie 1

Description: Processive motility of DDB-eGFP. Representative movie of DDB-eGFP complexes moving on a single microtubule (microtubule not shown). Minus end of the microtubule is located on the left. Movie plays 6xm faster than real time.

File name: Supplementary Movie 2

Description: Processive motility of KIF16B-eGFP. Representative movie of KIF16B-eGFP motors moving on a single microtubule (microtubule not shown). Minus end of the microtubule is located on the left. Movie plays 6x faster than real time.

File name: Supplementary Movie 3

Description: Minus-end directed motility of DDB-vesicles. Representative movie of DDB-vesicle (orange) moving on a single, polarity-marked microtubule (cyan). Minus end of the microtubule, represented by the brighter fluorescence signal, is located on the left. Movie plays 6x faster than real time.

File name: Supplementary Movie 4

Description: Plus-end directed motility of KIF16B-vesicles. Representative movie of KIF16B-vesicle (orange) moving on a single, polarity-marked microtubule (cyan). Minus end of the microtubule, represented by the brighter fluorescence signal, is located on the left. Movie plays 6x faster than real time.

File name: Supplementary Movie 5

Description: Minus-end, plus-end and reversing motility of DDB-KIF16B-vesicles. Representative movie of DDB-KIF16B-vesicle (orange) moving on a single, polarity-marked microtubule (cyan). Minus end of the microtubule, represented by the brighter fluorescence signal, is located on the left. Movie plays 6x faster than real time.

File name: Supplementary Movie 6

Description: DDB-KIF16B vesicle undergoing elongation during the paused states. Example movie of DDB-KIF16B-vesicle (orange) moving on a single, polarity-marked microtubule (cyan) and undergoing elongation during the paused states. Minus end of the microtubule, represented by the brighter fluorescence signal, is located on the left. Movie plays 6x faster than real time.
