# Supplementary material for: Vesicles driven by dynein and kinesin exhibit directional reversals without regulators
Source: Nat Commun. 2023 Nov 20;14:7532. doi: 10.1038/s41467-023-42605-8 (PMC10662051; doi:10.1038/s41467-023-42605-8)
Supplement: Supplementary file 10 — Reporting Summary [file 41467_2023_42605_MOESM10_ESM.pdf]

nature portfolio

Corresponding author(s): Santen and Diez

Last updated by author(s): Oct 5, 2023

Reporting Summary

Nature Portfolio wishes to improve the reproducibility of the work that we publish. This form provides structure for consistency and transparency in reporting. For further information on Nature Portfolio policies, see our Editorial Policies and the Editorial Policy Checklist.

Statistics

For all statistical analyses, confirm that the following items are present in the figure legend, table legend, main text, or Methods section.

n/a	Confirmed	
☐	☒	The exact sample size (*n*) for each experimental group/condition, given as a discrete number and unit of measurement
☐	☒	A statement on whether measurements were taken from distinct samples or whether the same sample was measured repeatedly
☐	☒	The statistical test(s) used AND whether they are one- or two-sided<br>*Only common tests should be described solely by name; describe more complex techniques in the Methods section.*
☒	☐	A description of all covariates tested
☐	☒	A description of any assumptions or corrections, such as tests of normality and adjustment for multiple comparisons
☐	☒	A full description of the statistical parameters including central tendency (e.g. means) or other basic estimates (e.g. regression coefficient) AND variation (e.g. standard deviation) or associated estimates of uncertainty (e.g. confidence intervals)
☐	☒	For null hypothesis testing, the test statistic (e.g. *F*, *t*, *r*) with confidence intervals, effect sizes, degrees of freedom and *P* value noted<br>*Give P values as exact values whenever suitable.*
☒	☐	For Bayesian analysis, information on the choice of priors and Markov chain Monte Carlo settings
☒	☐	For hierarchical and complex designs, identification of the appropriate level for tests and full reporting of outcomes
☒	☐	Estimates of effect sizes (e.g. Cohen's *d*, Pearson's *r*), indicating how they were calculated

Our web collection on statistics for biologists contains articles on many of the points above.

Software and code

Policy information about availability of computer code

Data collection	Microscopy images were captured using NIS elements software from Nikon.
Data analysis	Data were analyzed using custom scripts written in MATLAB and python

For manuscripts utilizing custom algorithms or software that are central to the research but not yet described in published literature, software must be made available to editors and reviewers. We strongly encourage code deposition in a community repository (e.g. GitHub). See the Nature Portfolio guidelines for submitting code & software for further information.

Data

Policy information about availability of data

All manuscripts must include a data availability statement. This statement should provide the following information, where applicable:

- Accession codes, unique identifiers, or web links for publicly available datasets
- A description of any restrictions on data availability
- For clinical datasets or third party data, please ensure that the statement adheres to our policy

The authors declare that the data used to generate all plots in this study are available in the source data file. The experimental and simulated tracks are available from the corresponding author upon reasonable request.

Research involving human participants, their data, or biological material

Policy information about studies with human participants or human data. See also policy information about sex, gender (identity/presentation), and sexual orientation and race, ethnicity and racism.

Reporting on sex and gender	Not applicable
Reporting on race, ethnicity, or other socially relevant groupings	Not applicable
Population characteristics	Not applicable
Recruitment	Not applicable
Ethics oversight	Not applicable

Note that full information on the approval of the study protocol must also be provided in the manuscript.

Field-specific reporting

Please select the one below that is the best fit for your research. If you are not sure, read the appropriate sections before making your selection.

☒ Life sciences ☐ Behavioural & social sciences ☐ Ecological, evolutionary & environmental sciences

For a reference copy of the document with all sections, see nature.com/documents/nr-reporting-summary-flat.pdf

Life sciences study design

All studies must disclose on these points even when the disclosure is negative.

Sample size	Sample size calculation for the experiment was not carried out a priori.
Data exclusions	Position-time tracks from vesicles were excluded if: 1) vesicles crossed a microtubule-microtubule junction as such vesicles would often pause and therefore erroneously alter the pausing frequency and duration analysis, 2) tracks were too short (<300 nm) for the segmentation algorithm to reliably identify runs and pauses, and 3) vesicles that slowed down upon colliding with another vesicle (moving or stationary) or a stationary bead. Pauses at the beginning and the end of a track were excluded as the activity state f the vesicle-bound motors were unclear before and after such pauses respectively. Reversals occurring at the end of a microtubule were excluded.
Replication	Data of dual- and single-motor vesicles were acquired from independent experiments performed over multiple days. Fresh vesicles and polarity-marked microtubules were prepared each day. Velocity analysis was first carried out on the two datasets individually and were only pooled similar trends were observed across the two datasets.
Randomization	Randomization was not applicable in our study because we always pooled all obtained data for evaluation.
Blinding	All experiments were performed by one researcher at any given point in time. Therefore blinding was not possible.

Reporting for specific materials, systems and methods

We require information from authors about some types of materials, experimental systems and methods used in many studies. Here, indicate whether each material, system or method listed is relevant to your study. If you are not sure if a list item applies to your research, read the appropriate section before selecting a response.

Materials & experimental systems

n/a	Involved in the study	
☐	☒	Antibodies
☐	☒	Eukaryotic cell lines
☒	☐	Palaeontology and archaeology
☒	☐	Animals and other organisms
☒	☐	Clinical data
☒	☐	Dual use research of concern
☒	☐	Plants

Methods

n/a	Involved in the study	
☒	☐	ChIP-seq
☒	☐	Flow cytometry
☒	☐	MRI-based neuroimaging

Antibodies

Antibodies used	Anti-dynein heavy chain antibody, Santa Cruz, sc-514579 (C-5), 1:2000;<br>Anti-dynactin p62 antibody, Santa Cruz, sc-55604 (H-12), 1:2000;<br>Anti-dynactin p50 antibody, Santa Cruz, sc-393389 (G-4),1:2000;<br>Anti-dynein intermediate chain antibody, Sigma Millipore, D5167, 1:5000;<br>Anti-mouse IgG-HRP antibody, Abcam, ab97023, 1:20000;<br>Anti-dynactin Arp1, Santa Cruz, sc-390632 (E-5), 1:2000;<br>Anti-dynactin p150, Santa Cruz, sc-135890 (12), 1:2000;
Validation	Anti-dynein heavy chain antibody: validated by manufacturer in human LNCaP cells for Western Blotting<br>Anti-dynactin p62 antibody: validated by manufacturer in human HeLa cells for Western Blotting<br>Anti-dynactin p50 antibody: validated by manufacturer in human MCF7 cells for Western Blotting<br>Anti-dynein intermediate chain antibody: validated by manufacturer in human HeLa cells for Western Blotting<br>Anti-mouse IgG-HRP antibody: validated by manufacturer on anti-mouse antibodies for Western Blotting<br>Anti-dynactin Arp1: validated by manufacturer in human HEK293 cells for Western Blotting<br>Anti-dynactin p150: validated by manufacturer in human HeLa cells for Western Blotting

Eukaryotic cell lines

Policy information about cell lines and Sex and Gender in Research

Cell line source(s)	Human Embryonic Kidney 293 cells, female, Commercial<br>Spodoptera frugiperda (Sf9+) cells, commercial
Authentication	None of the cell lines were authenticated.
Mycoplasma contamination	Cell lines were purchased from a commercial vendor. They have never tested positive for Mycoplasma.
Commonly misidentified lines<br>(See ICLAC register)	No commonly misidentified cell lines were used in the study..
